# A unified SVPWM fault tolerant control algorithm for single leg fault reconstruction topology of two-level inverter

**DOI:** 10.1038/s41598-024-59425-5

**Published:** 2024-04-17

**Authors:** Cai Zhiduan, Xu Lihao

**Affiliations:** School of Intelligent Manufacturing, Huzhou College, NO.1 Xueshi Rd., Huzhou, 313000 Zhejiang China

**Keywords:** Fault-tolerance, Voltage compensation, Inverter, Four-switch three-phase, Space vector pulse width modulation, Electrical and electronic engineering, Mechanical engineering

## Abstract

To improve the reliability of Two-level three phase voltage source inverters, a uniform fault tolerant strategy based on space vector pulse width modulation is proposed for different leg faults. The reconstructed topologies of inverters with different bridge arm faults are different, which makes the basic voltage vector phase of each reconstructed topology inconsistent, resulting in different calculations. Therefore, the coordinate transformation is applied to place the basic voltage vectors of each reconstructed topology on the synchronous stationary ***αβ*** coordinate system so that the calculations of the reconstructed topology under different bridge arm faults are unified, thus reducing the complexity of fault-tolerant control. Aiming at the three-phase current asymmetry caused by the neutral point voltage oscillation in inverter topology reconstruction, a transient compensation method of neutral point voltage offset for the ***α-***axis component of the reference voltage vector is introduced to suppress the adverse effects. The compensation method directly offers a neutral point voltage offset value after Clarke transformation and corrects the ***α-***axis component of the reference voltage vector, avoiding the integral calculation in the conventional voltage compensation algorithm. The correctness and effectiveness of the proposed fault-tolerant control strategy are verified experimentally.

## Introduction

**Figure 1:**
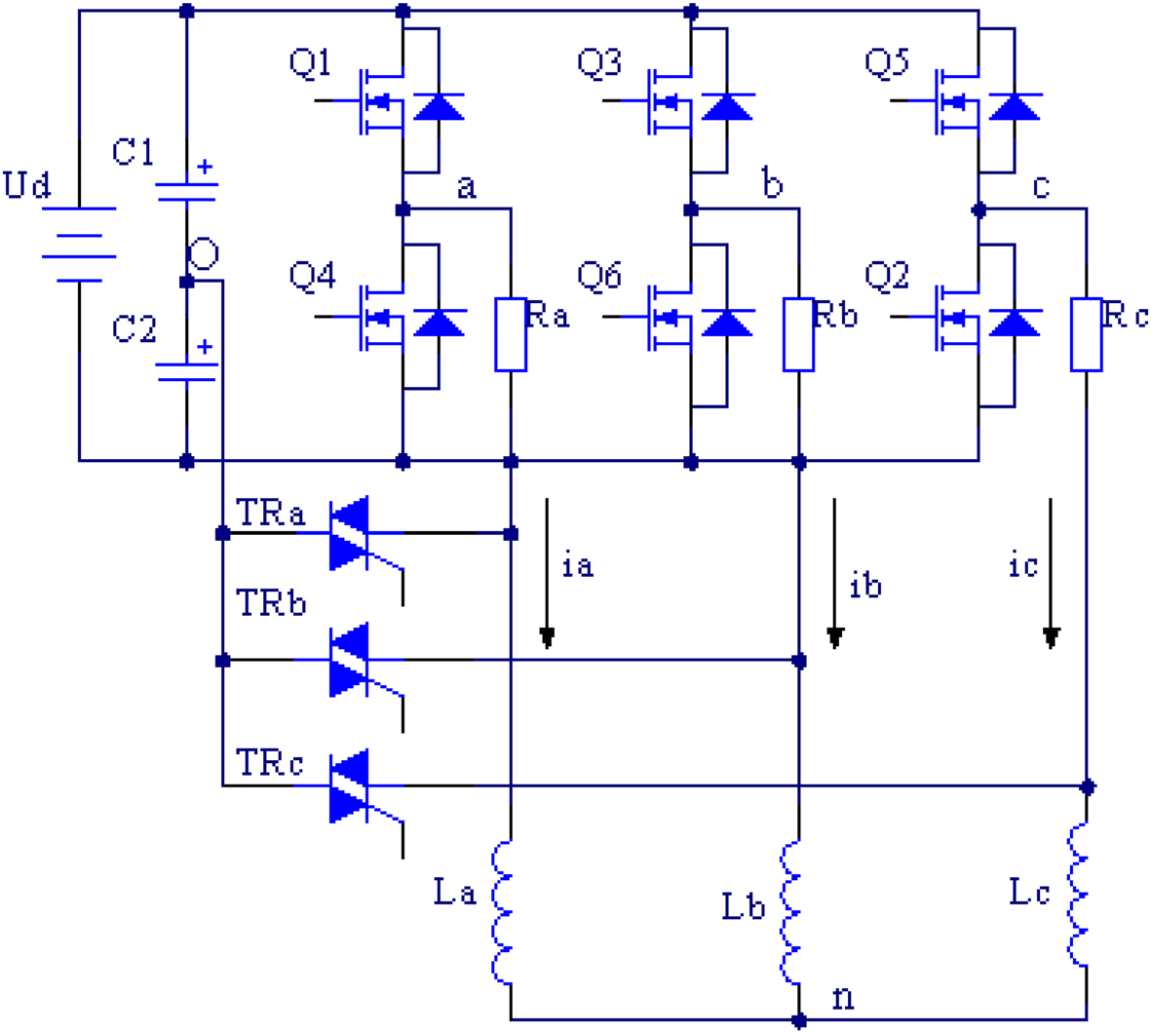
Fault-tolerant topology of a two-level inverter.

Two-level three-phase voltage source inverters are widely used in motor drives, active power filters, new energy grid connections and other occasions. Once the power transistor in an inverter malfunctions, the whole inverter will function abnormally or stop, which may cause catastrophic accidents on key occasions requiring continuous operation, such as in the aerospace industry, automotive industry, and energy industry, etc. In order to ensure the safe, reliable and continuous operation of inverters, it is of paramount necessity to set corresponding fault-tolerant control strategies for inverter faults. The fault-tolerant control of two-level three-phase voltage source inverters has been extensively studied^1–3^, including two reconstruction aspects: hardware topology and software control strategy. Various fault-tolerant reconfiguration topologies of three-phase voltage source inverters have been summarized in Refs.^1–3^. The fault-tolerant topology delineated in Fig. 1 is widely utilized because of its feature that the connection of three-phase load neutral points is not necessary and because there are very few redundant power transistors. This article adopts the fault-tolerant topology structure in Fig. 1. When the power transistor of a certain bridge arm fails, the corresponding faulty bridge arm is isolated by disconnecting the fast fuse Fa, Fb, or Fc; then, the load of the fault phase is connected to the midpoint of the two capacitors on the DC side by conducting bidirectional thyristors TRa, TRb, or TRc to achieve topology reconstruction.

**Figure 2:**
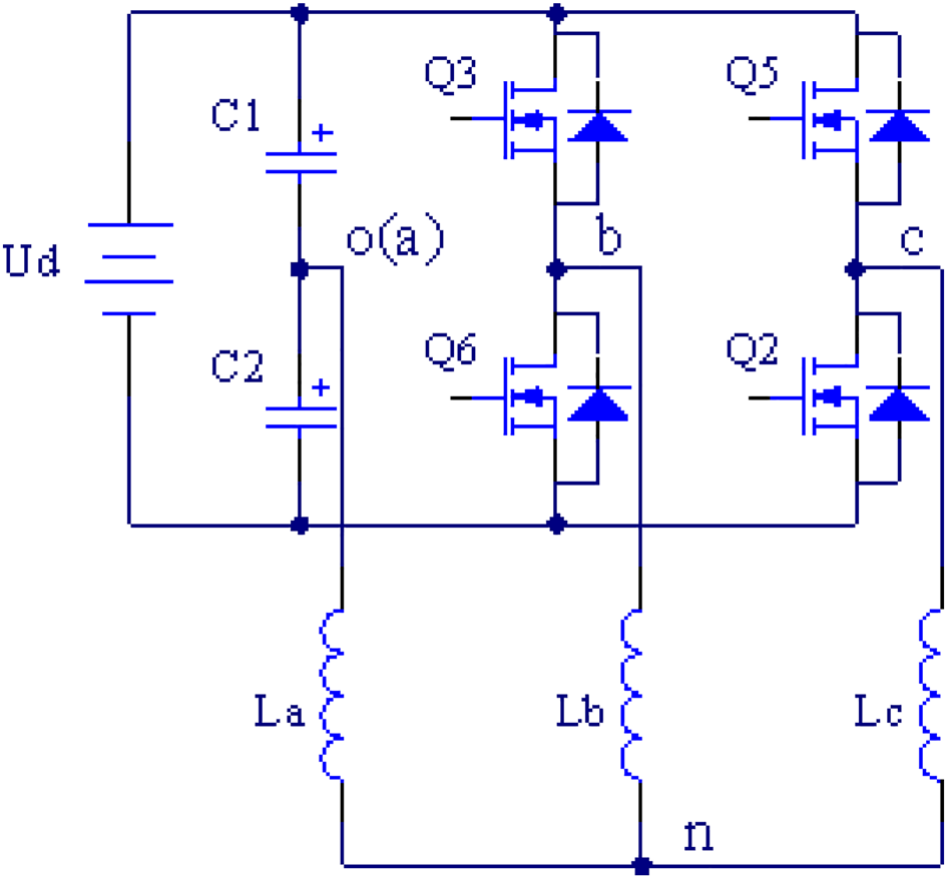
Reconfigured topology under leg A fault.

Figure 2 depicts the reconstructed topology after a fault in the A-phase bridge arm occurs. Due to the topology reconstruction, the appropriate control algorithm of the inverter also needs to be modified. Two-level three-phase voltage source inverters often apply two control methods: sinusoidal pulse width modulation (SPWM) and space vector pulse width modulation (SVPWM). The SVPWM owns a higher utilization rate of DC voltage and does not require triangular carriers, allowing it to be broadly employed^4–6^.

The main task of SVPWM is to synthesize the target voltage vector based on “Volt-Second” relation.

**Step 1** The sector of reference voltage vector is determined;

**Step 2** The appropriate basic voltage vector based on the sector is selected;

**Step 3** The target vector is synthesized by calculating the action time of basic voltage vector.

In motor control, active power filtering and other application fields, SVPWM fault-tolerant control algorithms for single leg faults were used in Refs.^7–26^. The above algorithms include vector sequence reconstruction of optimal basic voltage and action time adjustment of basic voltage vector, which attains good results. However, the above literature merely analyses SVPWM for fault reconstruction topology of a single phase bridge arm, rather than the other two-phase bridge arms. Due to the different fault-tolerant topologies of the inverter after the fault of different phase bridge arms, the non-zero basic voltage vectors corresponding to the fault-tolerant topology are also different, which leads to the inconsistent judgment of the target vector sector, the calculation of the action time of the basic voltage vector and the action order of the basic vector in the implementation process of SVPWM. The difficulty and computational complexity of fault-tolerant control process are greatly enhanced owing to the inconsistent SVPWM calculations for reconstructing the topology of inverters with different bridge arm faults. Therefore, if SVPWM is unified, the difficulty and complexity will be reduced. At present, the unified SVPWM algorithm has not been studied. The difficulty and complexity of SVPWM under different bridge arm faults is considered in Refs.^15,16^, and SVPWM calculation is optimized. According to the “Volt-Second” relation, the equivalence of the calculation formula describing basic voltage vector action time with different reconfiguration topologies is simply narrated in Ref.^15^. However, the calculation of basic voltage vector action time still requires complex calculations, such as trigonometric functions. Sector determination and calculation of basic voltage vector action time is simplified in Ref.^16^ by changing target vector synthesis formula; however, this method changes the phase sequence of the three-phase output power, which is not suitable for fault-tolerant control in motor drives, power grid connections or other applications.

In addition, since the neutral point of the capacitor at the DC side of inverter topology is directly connected to the load, the phase current passing through the capacitor will lead to voltage oscillation at the neutral point. The oscillation may shift the basic voltage vector, destroy SVPWM modulation, and ultimately aggravate the inverter output performance^18,23–26^. Consequently, the neutral point voltage oscillation of SVPWM needs to be compensated. In order to meet this requirement, the neutral point voltage offset needs to be obtained online. One method is direct detection by using a voltage sensor, which requires additional hardware. Another method is to integrate the existing phase current^23–25^. However, the integration not only complicates inverter control but also deteriorates compensation due to phase errors caused by integration time.

So as to solve the above two problems, a unified SVPWM algorithm is proposed for different bridge arm fault reconstruction topologies, which makes the sector determination and the calculation of basic vector action time consistent, as well as reduces the difficulty and complexity of the algorithm in fault-tolerant control process. In addition, after analyzing the essence of voltage oscillation, an instantaneous voltage offset compensation method is proposed through Clarke coordinate transformation, which mitigates the impact of DC-side midpoint voltage oscillation, and avoids integral operation in conventional compensation methods.

## Switching function model of the inverter reconfiguration topology

Taking the A-phase bridge arm fault as an example, the inverter reconfiguration topology is displayed in Fig. 2. $S_{a}$, $S_{b}$ and $S_{c}$ are defined to represent the switching states of three-phase bridge arm power transistors respectively. A value of “1” means that the upper transistor is connected and the lower one is closed, while a value of “0” means that the upper transistor is closed and the lower one is connected^17^. The description model of three-phase output voltage is established as follows:

1 $$ \left[ {\begin{array}{*{20}l} {u_{a0} } \hfill \\ {u_{b0} } \hfill \\ {u_{c0} } \hfill \\ \end{array} } \right] = \left[ {\begin{array}{*{20}c} 0 \\ {s_{b} u_{c1} + \left( {s_{b} - 1} \right)u_{c2} } \\ {s_{c} u_{c1} + \left( {s_{c} - 1} \right)u_{c2} } \\ \end{array} } \right] $$

where $u_{a0}$, $u_{b0}$ and $u_{c0}$ are the line voltages between the inverter output terminals and the midpoints of DC side capacitors and $u_{c1}$ and $u_{c2}$ are the voltages of $c_{1}$ and $c_{2}$, respectively.

Let $u_{an}$, $u_{bn}$ and $u_{{{\text{c}}n}}$ be phase voltages. According to the symmetry of three-phase system and the relationship between phase voltage and line voltage, (2) can be derived:

2 $$ \left[ {\begin{array}{*{20}l} {u_{an} } \hfill \\ {u_{bn} } \hfill \\ {u_{cn} } \hfill \\ \end{array} } \right] = \frac{{u_{c1} }}{3}\left[ {\begin{array}{*{20}l} { - s_{b} - s_{c} } \hfill \\ {2s_{b} - s_{c} } \hfill \\ {2s_{c} - s_{b} } \hfill \\ \end{array} } \right] + \frac{{u_{c2} }}{3}\left[ {\begin{array}{*{20}c} {2 - s_{b} - s_{c} } \\ {2s_{b} - s_{c} - 1} \\ {2s_{c} - s_{b} - 1} \\ \end{array} } \right] $$

With phase A as the reference phase, the voltage vector synthesis formula is as follows:

3 $$ u_{s} = \frac{2}{3}\left( {u_{an} + e^{{j\frac{2\pi }{3}}} u_{bn} + e^{{j\frac{4\pi }{3}}} u_{cn} } \right) $$

where in the synchronous stationary coordinate $\alpha \beta$, $u_{s}$ can be described as:

4 $$ u_{s} = u_{\alpha } + ju_{\beta } $$

Equation (2) can be converted to $\alpha \beta$ coordinates by Clarke transformation:

5 $$ \left[ {\begin{array}{*{20}c} {u_{\alpha } } \\ {u_{\beta } } \\ \end{array} } \right] = A\left[ {\begin{array}{*{20}c} {u_{an} } \\ {u_{bn} } \\ {u_{cn} } \\ \end{array} } \right] = \left[ {\begin{array}{*{20}c} {\left( { - \frac{{s_{b} + s_{c} }}{2}} \right)u_{c1} + \left( {1 - \frac{{s_{b} + s_{c} }}{2}} \right)u_{c2} } \\ {\left( {\frac{{\sqrt 3 (s_{b} - s_{c} )}}{2}} \right)u_{c1} + \left( {\frac{{\sqrt 3 (s_{b} - s_{c} )}}{2}} \right)u_{c2} } \\ \end{array} } \right] $$

where $$A = \frac{2}{3}\left[ {\begin{array}{*{20}c} 1 & { - \frac{1}{2}} & { - \frac{1}{2}} \\ 0 & {\frac{\sqrt 3 }{2}} & { - \frac{\sqrt 3 }{2}} \\ \end{array} } \right]$$ is the Clarke transformation matrix.

Considering voltage oscillation at the midpoint of the capacitor, let $\Delta u$ be the voltage offset, and then substitute $u_{c1} = \frac{1}{2}u_{dc} + {\Delta }u$ and $u_{c2} = \frac{1}{2}u_{dc} - {\Delta }u$ into (5) to obtain (6). Similarly, in the case of a B/C-phase bridge arm fault, the switching function models of inverter in $\alpha \beta$ coordinates can be established, which are (7) and (8), respectively:

6 $$ \left[ {\begin{array}{*{20}c} {u_{\alpha } } \\ {u_{\beta } } \\ \end{array} } \right] = \left[ {\begin{array}{*{20}c} {\frac{{u_{dc} }}{3}(1 - s_{b} - s_{c} ) - \frac{{2{\Delta }u}}{3}} \\ {\frac{{u_{dc} }}{\sqrt 3 }(s_{b} - s_{c} )} \\ \end{array} } \right] $$

7 $$ \left[ {\begin{array}{*{20}c} {u_{\alpha } } \\ {u_{\beta } } \\ \end{array} } \right] = \left[ {\begin{array}{*{20}c} {\frac{{u_{dc} }}{3}\left( {2s_{a} - s_{c} - \frac{1}{2}} \right) + \frac{{{\Delta }u}}{3}} \\ {\frac{{u_{dc} }}{\sqrt 3 }\left( {\frac{1}{2} - s_{c} } \right) - \frac{1}{\sqrt 3 }{\Delta }u} \\ \end{array} } \right] $$

8 $$ \left[ {\begin{array}{*{20}c} {u_{\alpha } } \\ {u_{\beta } } \\ \end{array} } \right] = \left[ {\begin{array}{*{20}c} {\frac{{u_{dc} }}{3}\left( {2s_{a} - s_{b} - \frac{1}{2}} \right) + \frac{{{\Delta }u}}{3}} \\ {\frac{{u_{dc} }}{\sqrt 3 }\left( { - \frac{1}{2} + s_{b} } \right) + \frac{1}{\sqrt 3 }{\Delta }u} \\ \end{array} } \right] $$

## SVPWM under different bridge arm faults

### The basic voltage vector of reconstructed topology

**Figure 3:**
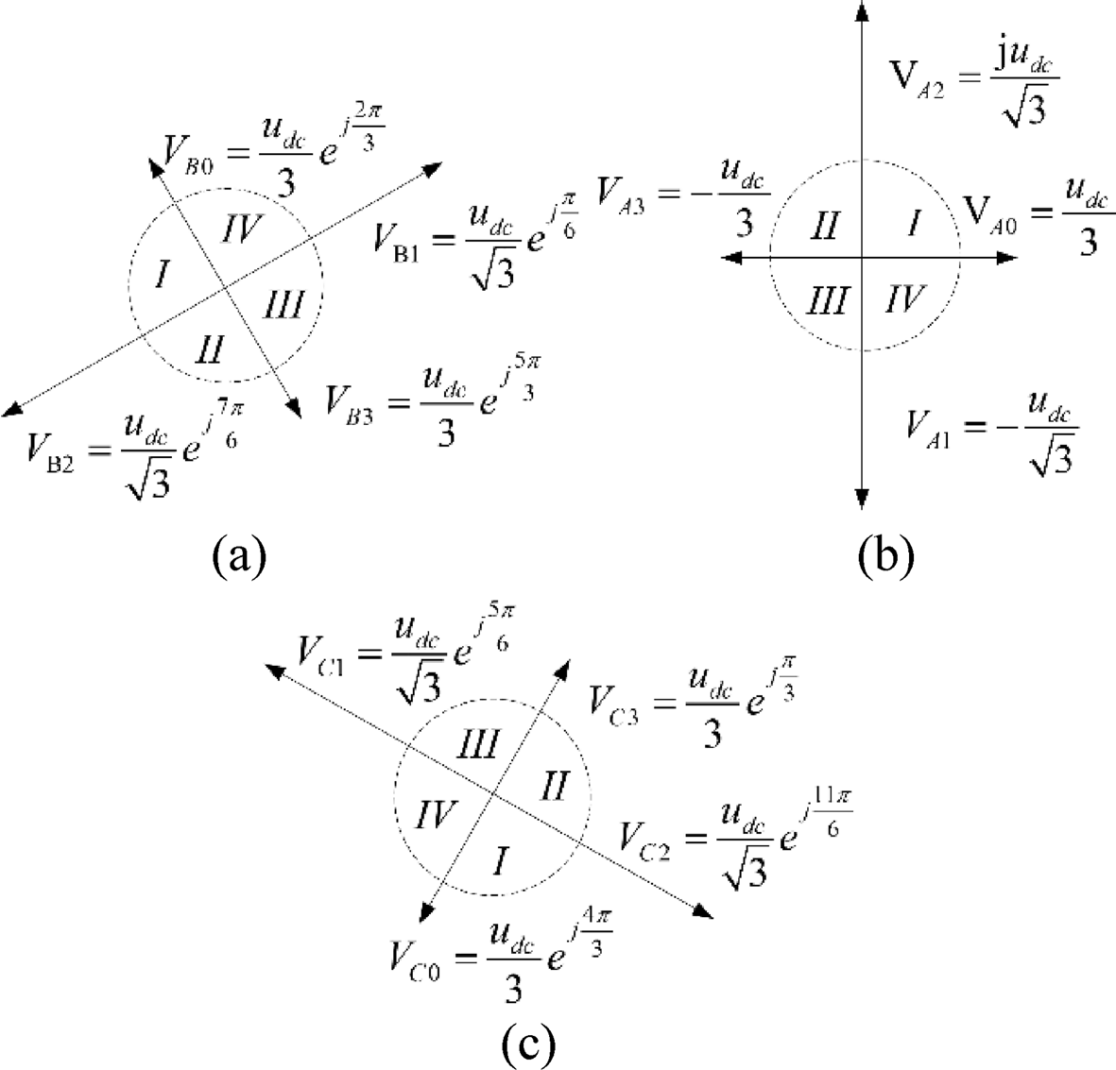
Distribution diagram of the basic voltage vectors: (**a**) phase A bridge arm fault; (**b**) phase B bridge arm fault; (**c**) phase C bridge arm fault.

For the convenience of analysis and description, ${\Delta }u$ is set to zero. Combining (6), (7) and (8), the basic voltage vector distribution graph of the reconstructed topology under different bridge arm faults in $\alpha \beta$ coordinate system can be plotted as Fig. 3^18–22^.

There exist only four nonzero basic voltage vectors in the inverter reconfiguration topology, and the phases of the four vectors are inconsistent under different bridge arm faults, which results in the differences in the judgment of target voltage vector sector, the selection of basic vector and the calculation of action time during SVPWM. The judgment of sector and calculation of basic vector action time are discussed in detail in Refs.^18–20^. The conclusion of the above documents is that since the basic voltage vector of the inverter reconstruction topology is distributed on two coordinate axes when an A-phase bridge arm fault occurs, the sector judgment and the action time of the basic vector are relatively simpler, while the calculations of the other two faults are more complex.

**Figure 4:**
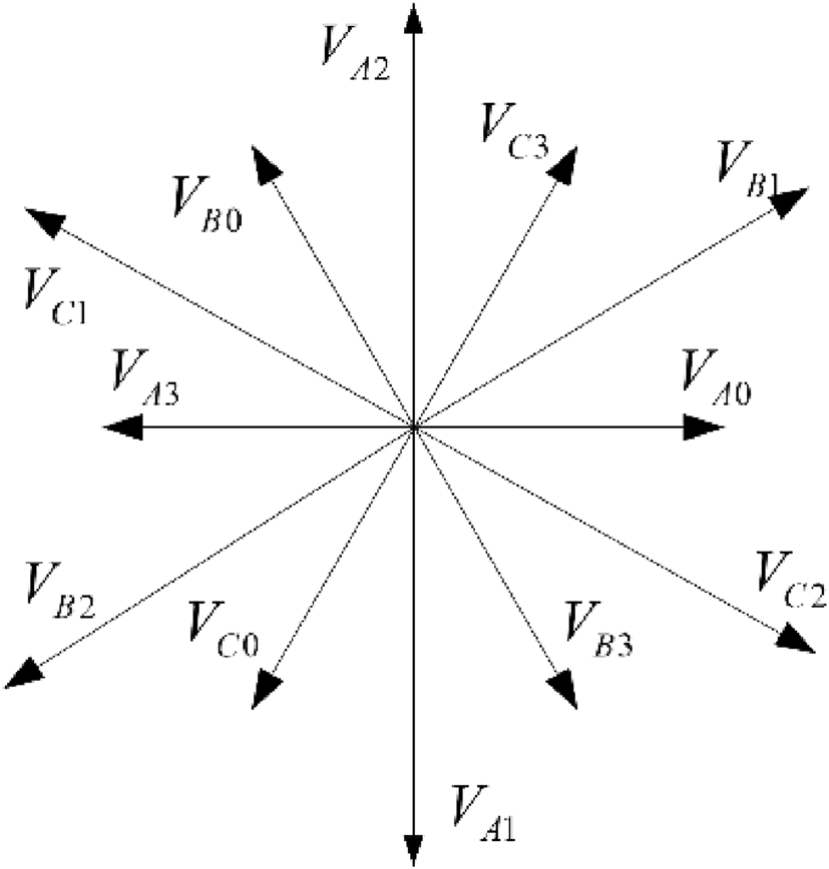
Voltage vectors under different leg faults.

Considering Fig. 3, 12 basic voltage vectors of the reconstructed topology under three different bridge arm faults are drawn in the same coordinate system, as exhibited in Fig. 4. It can be seen that the vector space is divided into 12 sectors by 12 basic voltage vectors, which is twice the division of 6 sectors under normal conditions.

### Coordinate transformation of basic voltage vector

Since the power transistors on three bridge arms are all possible to fail, 12 basic voltage vectors may appear in the fault-tolerant control process. There are 12 judgment criteria for the 12 sectors in Fig. 4, and 12 calculation methods for the action time of the basic vector, which greatly imposes computational burden and fault tolerance difficulty. To update inconsistent SVPWMs of inverter reconfiguration topology under different bridge arm faults, basic voltage vectors are rotated to the same distribution form through coordinate transformation under the principle of keeping the synthetic reference vector unchanged; thus, the SVPWMs can remain the same.

According to the “Volt-Second” relation in SVPWM vector synthesis, Eq. (2) is exported:

9 $$ u_{ref} T_{s} = u_{x} T_{x} + u_{y} T_{y} + u_{0} T_{0} $$

Referring to Eqs. (4), (9) can be written as an expression in $\alpha \beta$ coordinates:

10 $$ \left[ {\begin{array}{*{20}c} {u_{ref\alpha } } \\ {u_{ref\beta } } \\ \end{array} } \right]T_{s} = \left[ {\begin{array}{*{20}c} {u_{x\alpha } } \\ {u_{x\beta } } \\ \end{array} } \right]T_{x} + \left[ {\begin{array}{*{20}c} {u_{y\alpha } } \\ {u_{y\beta } } \\ \end{array} } \right]T_{y} + \left[ {\begin{array}{*{20}c} {u_{0\alpha } } \\ {u_{0\beta } } \\ \end{array} } \right]T_{0} $$

where $u_{ref}$ is the reference vector; $u_{x}$ and $u_{y}$ are the two basic voltage vectors; $u_{0}$ is the zero vector; $T_{s}$ is the sampling period; $T_{x}$ and $T_{y}$ are the action times of each basic vector; and T0 is the action time of zero vector, $T_{s} = T_{x} + T_{y} + T_{0}$. If both sides of (10) are multiplied by the coordinate transformation matrix, the equation still holds. That is, coordinate transformation is carried out simultaneously between the target voltage vector and the basic voltage vector, which can ensure that the selection of the basic voltage vector and the action time value remain unchanged before and after the transformation, and so does SVPWM.

Since the SVPWM of reconstructing topology in case of a phase A bridge arm fault is the simplest^18–20^, the voltage vectors of phase B and C bridge arm faults in Fig. 3b,c can be rotated through coordinate transformation, to be consistent with Fig. 3a. Taking the phase B bridge arm fault as an example, the coordinate rotation transformation matrix is calculated. If phase B is taken as the reference phase, the voltage vector synthesis formula is:

11 $$ \hat{u}_{s} = \frac{2}{3}\left( {u_{bn} + e^{{j\frac{2\pi }{3}}} u_{an} + e^{{j\frac{4\pi }{3}}} u_{cn} } \right) $$

It is obvious that the formats of (11) and (3) are similar.

As the three-phase bridge arms are symmetrical, combined with the basic voltage vector calculation method, the distribution of the basic voltage vector of phase B bridge arm fault is the same as that in Fig. 3a; that is, the four basic voltage vectors are distributed on the $\alpha \beta$ synchronous stationary coordinate system. The Clarke transformation is implemented to convert (11) to $\alpha \beta$ coordinates:

12 $$ \left[ {\begin{array}{*{20}c} {\hat{u}_{\alpha } } \\ {\hat{u}_{\beta } } \\ \end{array} } \right] = B\left[ {\begin{array}{*{20}c} {u_{an} } \\ {u_{bn} } \\ {u_{cn} } \\ \end{array} } \right] $$

where $\hat{u}_{s} = \hat{u}_{\alpha } + j\hat{u}_{\beta }$ and B is the transformation matrix. Taking the Euler formula into account, combined with (11) and (12), the Clarke transformation matrix B is:

13 $$ B = \frac{2}{3}\left[ {\begin{array}{*{20}c} { - \frac{1}{2}} & 1 & { - \frac{1}{2}} \\ {\frac{\sqrt 3 }{2}} & 0 & { - \frac{\sqrt 3 }{2}} \\ \end{array} } \right] $$

Since the vector synthesis of (11) takes phase B as the reference phase, the phase sequence is changed compared with (3), but the unified fault tolerance effect is not achieved. To avoid changing the phase sequence, set:

14 $$ \hat{B}A = B $$

Substituting matrices A and B in (5) and (13) into (14), (15) is integrated:

15 $$ \hat{B} = \left[ {\begin{array}{*{20}c} { - \frac{1}{2}} & {\frac{\sqrt 3 }{2}} \\ {\frac{\sqrt 3 }{2}} & \frac{1}{2} \\ \end{array} } \right] $$

where $\hat{B}$ is the coordinate rotation transformation matrix that distributes the basic voltage vector of phase $B$ on the $\alpha \beta$ static coordinate system without changing phase sequence.

Likewise, the coordinate rotation transformation matrix $\hat{C}$ in the case of a phase $C$ fault is:

16 $$ \hat{C} = \left[ {\begin{array}{*{20}c} { - \frac{1}{2}} & { - \frac{\sqrt 3 }{2}} \\ {\frac{\sqrt 3 }{2}} & { - \frac{1}{2}} \\ \end{array} } \right] $$

### Unified SVPWM algorithm

The specific implementation process of the unified SVPWM algorithm for different arm faults can be mainly divided into the following four steps:

**Step 1.** Coordinate transformation of voltage vector.

**Figure 5:**
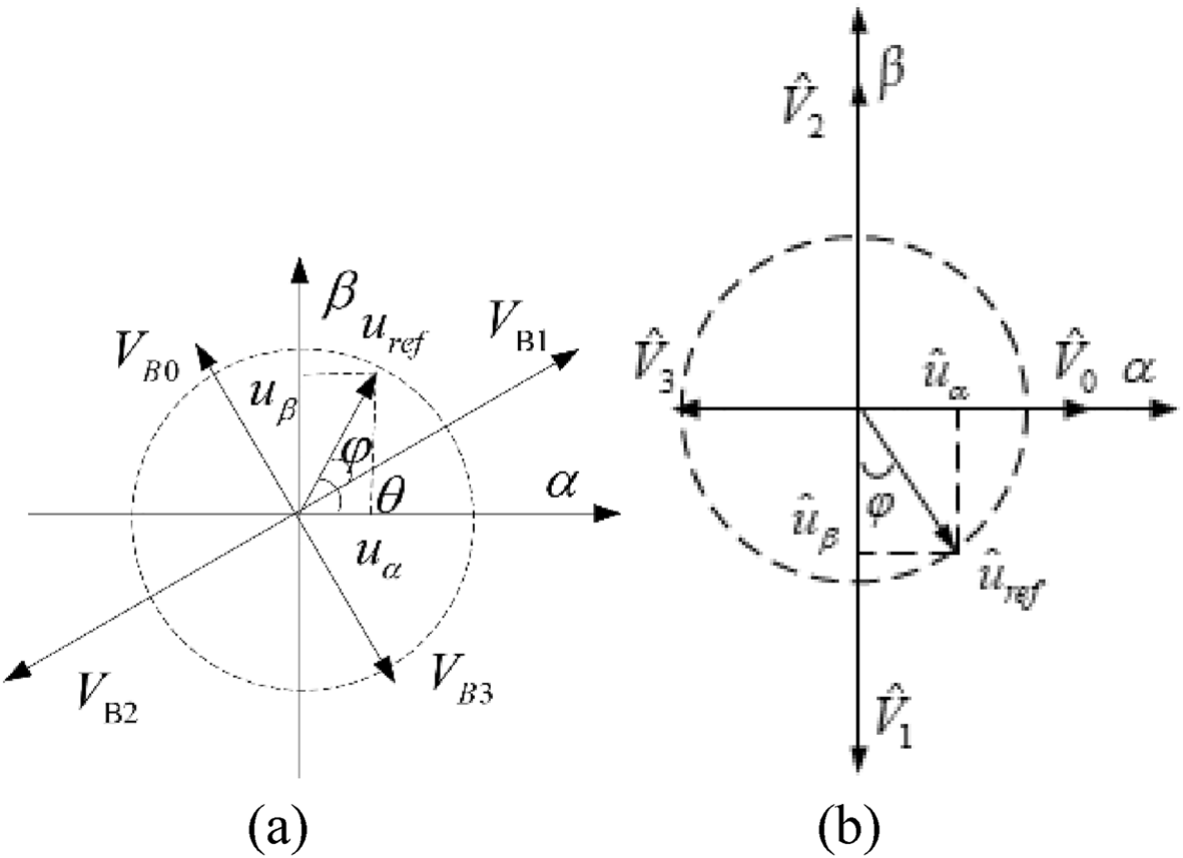
Vector synthesis of the reference voltage: (**a)** before coordinate transformation; (**b**) after coordinate transformation.

For phase a $B$ or phase $C$ bridge arm fault, the reference vector $u_{ref}$ and the basic voltage vector are multiplied by the corresponding coordinate transformation matrix $\hat{B}$ or $\hat{C}$ to obtain the transformed reference vector value $\hat{u}_{ref}$ and the basic voltage vector. Figure 5 reveals the vector synthesis before and after the coordinate transformation of voltage vector in the case of a phase $B$ bridge arm fault. Figure 5a,b are vector composite graphs before and after coordinate transformation respectively. The vector distribution of phase $C$ is similar. The four non-zero voltage basic vectors after unification are collectively referred to as $\hat{V}_{0}$, $\hat{V}_{1}$, $\hat{V}_{2}$,$\hat{V}_{3}$.

**Step 2.** Reference vector sector judgement.

After coordinate rotation transformation, the basic voltage vectors of different bridge arms are uniformly rotated to the axis in the $\alpha \beta$ coordinate system in Fig. 5b. Consequently, the judgement of the sector merely requires focusing on the symbols $\hat{u}_{ref\alpha }$ and $\hat{u}_{ref\beta }$ to determining where the reference vector is located after transformation.

Table 1 offers a comparison of sector judgment before and after coordinate transformation, where:

17 $$ \theta = \arcsin (u_{\alpha } /u_{\beta } ) $$

**Table 1:** Comparison of the sector judgment methods.

Sector	Conventional method		Improved method
	Phase B fault	Phase C fault	
I	$\theta \in \left[ {\frac{2\pi }{3},\frac{7\pi }{6}} \right]$	$\theta \in \left[ {\frac{4\pi }{3},\frac{11\pi }{6}} \right]$	$\hat{u}_{ref\alpha } \ge 0$ $\hat{u}_{ref\beta } \ge 0$
II	$\theta \in \left( {\frac{7}{6},\frac{5\pi }{3}} \right]$	$\theta \in \left[ {0,\frac{\pi }{3}} \right] \cup \left( {\frac{11\pi }{6},2\pi } \right]$	$\hat{u}_{ref\alpha } < 0$ $\hat{u}_{ref\beta } \ge 0$
III	$\theta \in \left( {\frac{5\pi }{3},2\pi } \right] \cup \left[ {0,\frac{\pi }{6}} \right]$	$\theta \in \left( {\frac{\pi }{3},\frac{5\pi }{6}} \right]$	$\hat{u}_{ref\alpha } \le 0$ $\hat{u}_{ref\beta } < 0$
IV	$\theta \in \left( {\frac{\pi }{6},\frac{2\pi }{3}} \right)$	$\theta \in \left( {\frac{5\pi }{6},\frac{4\pi }{3}} \right)$	$\hat{u}_{ref\alpha } > 0$ $\hat{u}_{ref\beta } < 0$

Table 1 implies that when using conventional sector judgement method, it is necessary to obtain the phase angle $\theta$ of the reference vector through an inverse trigonometric function^1,2,8^. Due to the accuracy limitation of the controller, these trigonometric functions not only increase the amount of calculation but also have a large error, which worsens SVPWM performance.

**Step 3.** Calculation of the action time of basic voltage vector.

According to the basic voltage vector in Fig. 5b and the “Volt-Second” relation of (9), the action time of the basic voltage vector can be obtained. Table 2 provides a comparison of calculation methods for the action time of basic voltage vector before and after coordinate transformation, where $\hat{T}_{0}$, $\hat{T}_{1}$, $\hat{T}_{2}$, $\hat{T}_{3}$ are the action duration of four non-zero basic voltage vectors respectively.

**Table 2:** Actuation duration of basic voltage vectors.

Action time	Conventional method	Improved method
$\hat{T}_{0}$	$\frac{{3\left| {u_{ref} } \right|\sin \varphi }}{{u_{dc} }}T_{s}$	$\frac{{3\hat{u}_{ref\alpha } }}{{u_{dc} }}T_{s}$
$\hat{T}_{1}$	$\frac{{\sqrt 3 \left| {u_{ref} } \right|\cos \varphi }}{{u_{dc} }}T_{s}$	$- \frac{{\sqrt 3 \hat{u}_{ref\beta } }}{{u_{dc} }}T_{s}$
$\hat{T}_{2}$	$\frac{{\sqrt 3 \left| {u_{ref} } \right|\cos \varphi }}{{u_{dc} }}T_{s}$	$\frac{{\sqrt 3 \hat{u}_{ref\beta } }}{{u_{dc} }}T_{s}$
$\hat{T}_{3}$	$\frac{{3\left| {u_{ref} } \right|\sin \varphi }}{{u_{dc} }}T_{s}$	$- \frac{{3\hat{u}_{ref\alpha } }}{{u_{dc} }}T_{s}$

Table 2 draws the conclusion that after coordinate transformation, the action time of the basic voltage vector only needs arithmetic, while the conventional method needs irrational numbers and trigonometric functions, and the calculation burden is greater^1,2,8^.

**Step 4.** The action sequence of basic voltage vector.

The basic voltage vector in SVPWM can be divided into five segments. A detailed introduction is elaborated in Refs.^11,19,24^.

## The compensation of the DC-side neutral point voltage

As exhibited in Fig. 2, after the topology is reconstructed, the phase current passes through the DC side capacitors $C_{1}$ and $C_{2}$, causing the neutral point voltage to shift. The neutral point voltage offset is set as ${\Delta }u$; then, $u_{c1} = u_{dc} /2 + {\Delta }u$, $u_{c1} = u_{dc} /2 - {\Delta }u$, ${\Delta }u = (u_{c1} - u_{c2} )/2$. From (6), (7) and (8), it can be concluded that the neutral point voltage offset also affects the basic voltage vector, making the three-phase output asymmetric^18,24–26^, which reduces the DC voltage utilization rate and the inverter performance. To remedy the above defects, the neutral point voltage offset can be compensated during SVPWM. From (6), it can be discovered that in the case of phase A bridge arm fault, there is $- 2/3{\Delta }u$ offset of the $\alpha$ component of the basic voltage vector generated by the neutral point voltage offset, but there is no effect on the $\beta$ component. Therefore, $u_{\alpha }$ is the only component to be compensated. Equations (7) and (8) indicate that when phase B and C bridge arms fail, the neutral point voltage offset affects both $\alpha$ and $\beta$ at the same time, and it is essential to compensate both $u_{\alpha }$ and $u_{{_{\beta } }}$. Since this paper adopts a unified SVPWM algorithm for B and C phase bridge arm faults, when faults occur, only the $\alpha$ component needs to be compensated. Therefore, the unified SVPWM fault-tolerant control method proposed in this paper is also conducive to simplifying the voltage compensation.

Since in the reference vector synthesis of the reconstructed topology, the zero vector can be equivalent to two opposite basic vectors^18,24–26^, the vector composition (10) can be written as follows:

18 $$ \left[ {\begin{array}{*{20}c} {u_{ref\alpha } } \\ {u_{ref\beta } } \\ \end{array} } \right]T_{s} = \left[ {\begin{array}{*{20}c} {u_{x\alpha } } \\ {u_{x\beta } } \\ \end{array} } \right]T_{x} + \left[ {\begin{array}{*{20}c} {u_{y\alpha } } \\ {u_{y\beta } } \\ \end{array} } \right]T_{y} + \left[ {\begin{array}{*{20}c} {u_{x\alpha } + u_{x - \alpha } } \\ {u_{x\beta } + u_{x - \beta } } \\ \end{array} } \right]\frac{{T_{0} }}{2} $$

where $u_{x - \alpha }$ and $u_{x - \beta }$ are the $\alpha$ and $\beta$ axes components of the vector opposite to $u_{x}$, respectively. Because of an offset of $- 2/3{\Delta }u$ in the $\alpha$ axis component, the actual synthesized vector is:

19 $$ \begin{gathered} \left[ {\begin{array}{*{20}c} {u_{ref\alpha }^{\prime } } \\ {u_{ref\beta }^{\prime } } \\ \end{array} } \right]T_{s} = \left[ {\begin{array}{*{20}c} {u_{x\alpha } - \frac{2}{3}{\Delta }u} \\ {u_{x\beta } } \\ \end{array} } \right]T_{x} + \left[ {\begin{array}{*{20}c} {u_{y\alpha } - \frac{2}{3}{\Delta }u} \\ {u_{y\beta } } \\ \end{array} } \right]T_{y} \hfill \\ + \left[ {\begin{array}{*{20}c} {u_{x\alpha } - \frac{2}{3}{\Delta }u + u_{x - \alpha } - \frac{2}{3}{\Delta }u} \\ {u_{x\beta } + u_{x - \beta } } \\ \end{array} } \right]\frac{{T_{0} }}{2} \hfill \\ \end{gathered} $$

$T_{s} = T_{1} + T_{2} + T_{0}$, Eq. (19) can be written as:

20 $$ \left[ {\begin{array}{*{20}c} {u_{ref\alpha }^{\prime } } \\ {u_{ref\beta }^{\prime } } \\ \end{array} } \right]T_{s} = \left[ {\begin{array}{*{20}c} {u_{ref\alpha } } \\ {u_{ref\beta } } \\ \end{array} } \right]T_{s} - \left[ {\begin{array}{*{20}c} {\frac{2}{3}{\Delta }u} \\ 0 \\ \end{array} } \right]T_{s} $$

i.e.

21 $$ \left[ {\begin{array}{*{20}c} {u_{ref\alpha }^{\prime } + \frac{2}{3}{\Delta }u} \\ {u_{ref\beta }^{\prime } } \\ \end{array} } \right]T_{s} = \left[ {\begin{array}{*{20}c} {u_{ref\alpha } } \\ {u_{ref\beta } } \\ \end{array} } \right]T_{s} $$

Equation (21) reveals that by compensating $2/3{\Delta }u$ for $\alpha$ axis component of the actual reference voltage vector, the impact of voltage oscillation on the output of the inverter can be eliminated. The key to compensation is to obtain the voltage offset ${\Delta }u$. The three-phase current is defined as $i_{a} = I_{m} \cos (\omega t)$, $i_{b} = I_{m} \cos \left( {\omega t - \frac{2\pi }{3}} \right)$ and $i_{c} = I_{m} \cos \left( {\omega t + \frac{2\pi }{3}} \right)$. When the phase A bridge arm fails, the electric potential offset is ${\Delta }u = \frac{1}{C}\smallint \frac{1}{2}i_{a} dt$^20^, and $i_{a}$ is substituted to obtain:

22 $$ \Delta u = \frac{1}{2C\omega }I_{m} \sin (\omega t) $$

Equation (22) suggests that the conventional method for calculating voltage offset ${\Delta }u$ requires trigonometric function operation, which is complex. In this paper, the algorithm is improved by using the transient value of three-phase current. The voltage offset is calculated through arithmetic instead of integral. The specific method is as follows:

When the phase A bridge arm fails, the three-phase current is processed by Clarke transform:

23 $$ \left[ {\begin{array}{*{20}c} {i_{\alpha } } \\ {i_{\beta } } \\ \end{array} } \right] = A\left[ {\begin{array}{*{20}c} {i_{a} } \\ {i_{b} } \\ {i_{c} } \\ \end{array} } \right] = \left[ {\begin{array}{*{20}c} {I_{m} \cos \omega t} \\ {I_{m} \sin \omega t} \\ \end{array} } \right] $$

$i_{\beta } = I_{m} \sin (\omega t)$ is obtained from (23) and the offset from (22) is:

24 $$ {\Delta }u = \frac{1}{2C\omega }i_{\beta } $$

When the phase B bridge arm fails, ${\Delta }u = \frac{1}{C}\smallint \frac{1}{2}i_{b} dt$, and:

25 $$ \Delta u = \frac{1}{2C\omega }I_{m} \sin \left( {\omega t - \frac{2\pi }{3}} \right) $$

The three-phase current as shown in (26):

26 $$ \left[ {\begin{array}{*{20}c} {i_{\alpha } } \\ {i_{\beta } } \\ \end{array} } \right] = A\left[ {\begin{array}{*{20}c} {i_{b} } \\ {i_{a} } \\ {i_{c} } \\ \end{array} } \right] = \left[ {\begin{array}{*{20}c} {I_{m} \cos \left( {\omega t - \frac{2\pi }{3}} \right)} \\ {I_{m} \sin \left( {\omega t + \frac{\pi }{3}} \right)} \\ \end{array} } \right] $$

$i_{\beta } = I_{m} \sin \left( {\omega t + \frac{\pi }{3}} \right)$ is derived from (26). Since $i_{\beta } = I_{m} \sin \left( {\omega t - \frac{2\pi }{3} + \pi } \right)$, it is further simplified as follows:

27 $$ i_{\beta } = - I_{m} \sin \left( {\omega t - \frac{2\pi }{3}} \right) $$

Bringing (27) into (25), the offset is:

28 $$ {\Delta }u = - \frac{1}{2C\omega }i_{\beta } $$

Accordingly, when the phase C bridge arm fails, it is as follows:

29 $$ \Delta u = \frac{1}{2C\omega }I_{m} \sin \left( {\omega t + \frac{2\pi }{3}} \right) $$

The three-phase current is converted as shown in the following (30):

30 $$ \left[ {\begin{array}{*{20}c} {i_{\alpha } } \\ {i_{\beta } } \\ \end{array} } \right] = A\left[ {\begin{array}{*{20}c} {i_{c} } \\ {i_{a} } \\ {i_{b} } \\ \end{array} } \right] = \left[ {\begin{array}{*{20}c} {I_{m} \cos \left( {\omega t + \frac{2\pi }{3}} \right)} \\ { - I_{m} \sin \left( {\omega t - \frac{\pi }{3}} \right)} \\ \end{array} } \right] $$

$i_{\beta } = - I_{m} sin\left( {\omega t - \frac{\pi }{3}} \right)$, namely,$i_{\beta } = - I_{m} \sin \left( {\omega t + \frac{2\pi }{3} - \pi } \right)$ can be obtained, and the formula is further processed as follows:

31 $$ i_{\beta } = I_{m} \sin \left( {\omega t + \frac{2\pi }{3}} \right) $$

32 $$ {\Delta }u = \frac{1}{2C\omega }i_{\beta } $$

## Simulation and experiment

**Figure 6:**
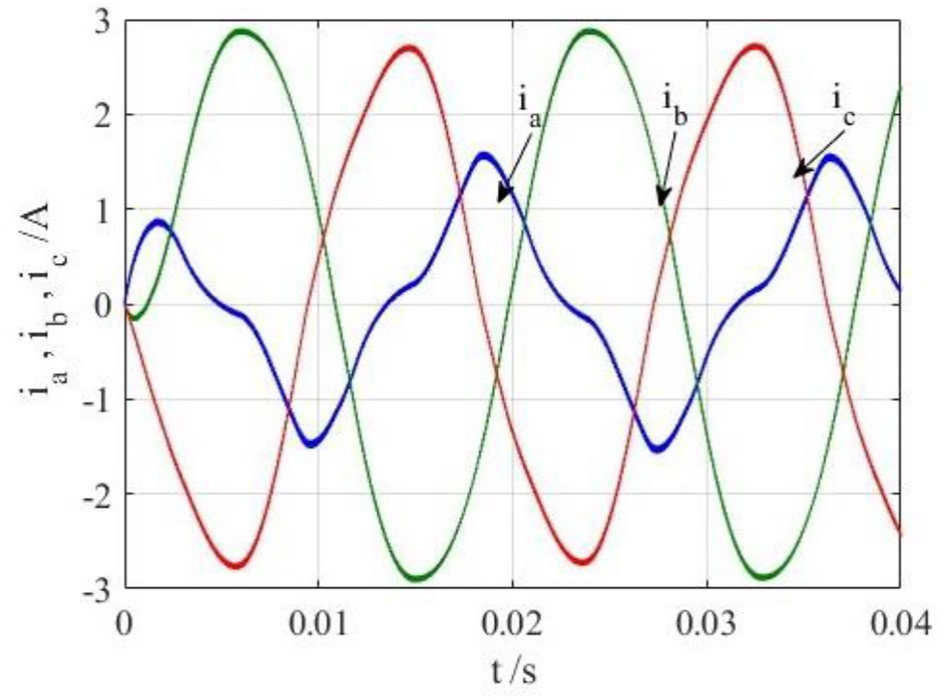
Simulated current waveforms without fault tolerant control algorithm.

**Figure 7:**
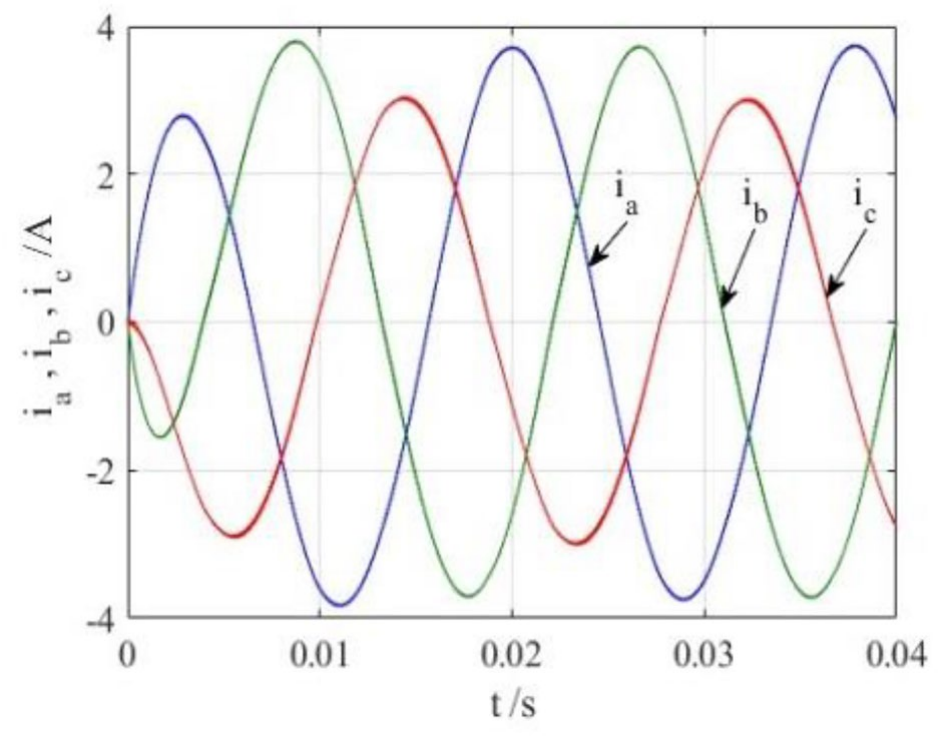
Simulated current waveforms without compensation.

**Figure 8:**
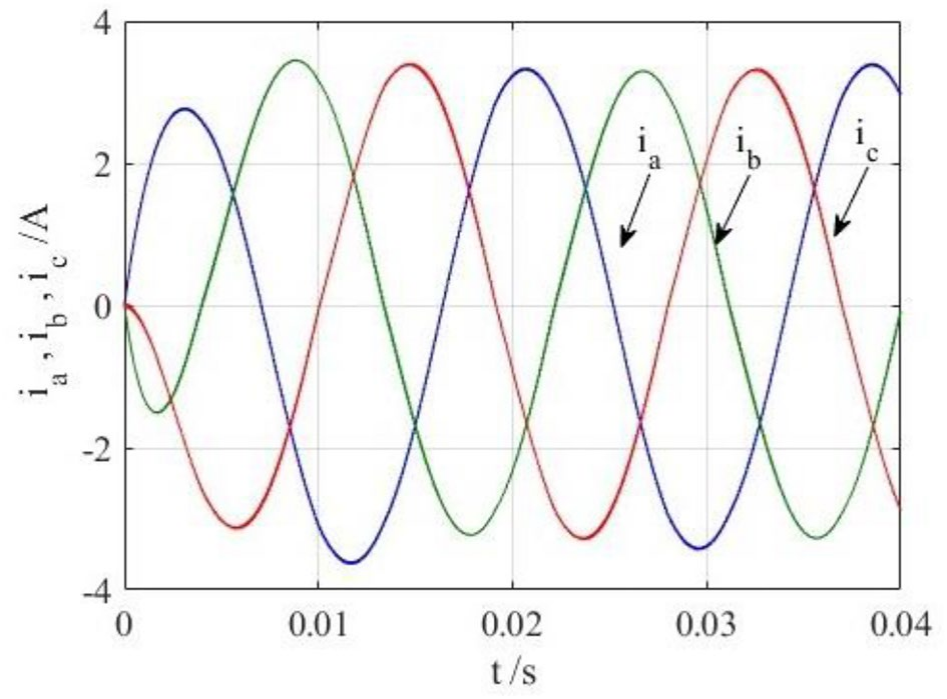
Simulated current waveforms using fault tolerant control algorithm under a phase A fault.

**Figure 9:**
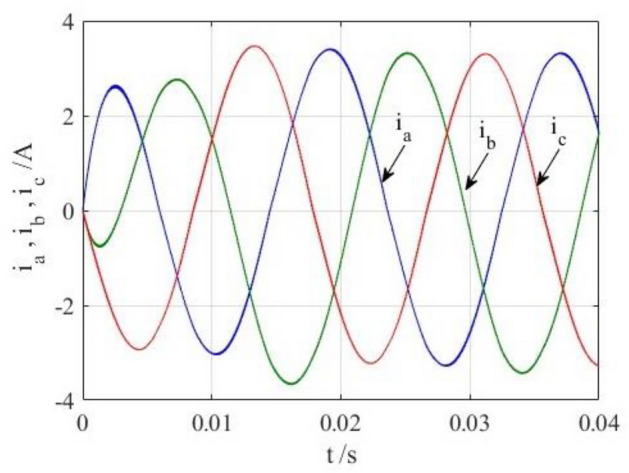
Simulated current waveforms using fault tolerant control algorithm under a phase B fault.

**Figure 10:**
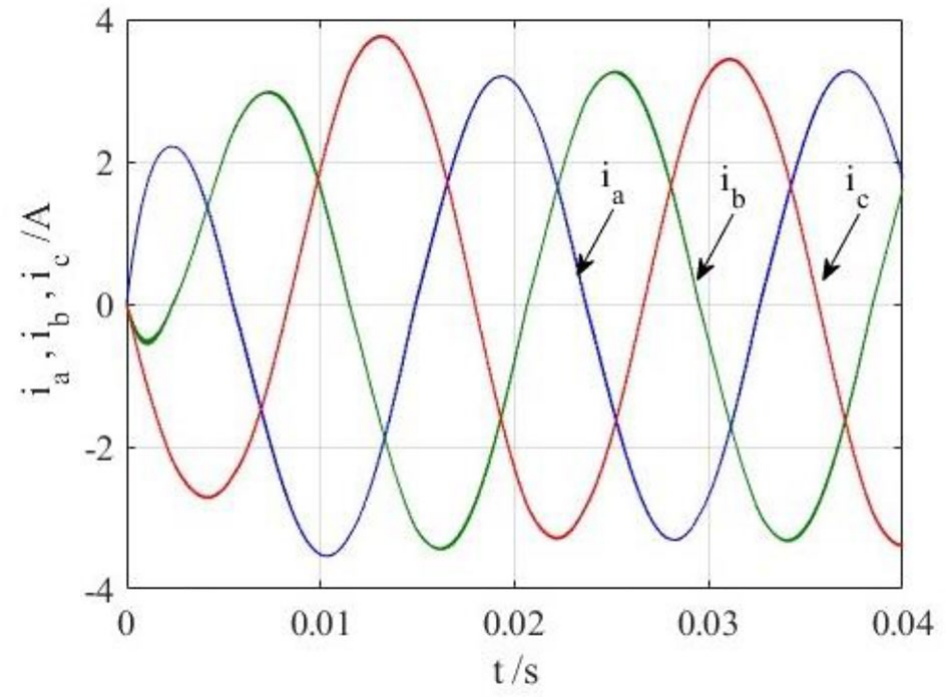
Simulated current waveforms using fault tolerant control algorithm under a phase C fault.

To verify the correctness of the fault-tolerant control strategy proposed in this paper, Simulink is utilized for simulation analysis. The simulation parameters are as follows: DC side voltage $U_{dc} = 48V$, DC side capacity $C_{1} = C_{2} = 1000uF$, load is three-phase resistance-inductance, the inductance of load is $L = 5.2mH$, the equivalent resistance is $R = 3.2{\Omega }$, the switching device is a MOSFET, and switching frequency is 14 kHz. The simulation results are displayed in Figs. 6, 7, 8, 9 and 10. Figure 6 depicts the current when SVPWM algorithm is not corrected after a fault occurs in the A-phase bridge arm. The three-phase current is seriously asymmetric and distorted due to the lack of a fault-tolerant control algorithm. Figure 7 illustrates the current when the “five segment” SVPWM fault-tolerant control algorithm is adopted, but the capacitor neutral point voltage offset is not compensated. It is evident that the three-phase current has better performance than that in Fig. 6 due to the appropriate correction, but the symmetry is still poor due to the lack of voltage offset compensation. Further, as in Fig. 8, voltage offset compensation method is adopted, and the three-phase current symmetry is greatly improved. Figures 9 and 10 show the phase current of the inverter after adopting the unified SVPWM and voltage compensation fault-tolerant control algorithm proposed in this paper when phases B and C fail respectively. The unified SVPWM algorithm can achieve good fault-tolerant effect and the three-phase current sequence remains unchanged.

**Figure 11:**
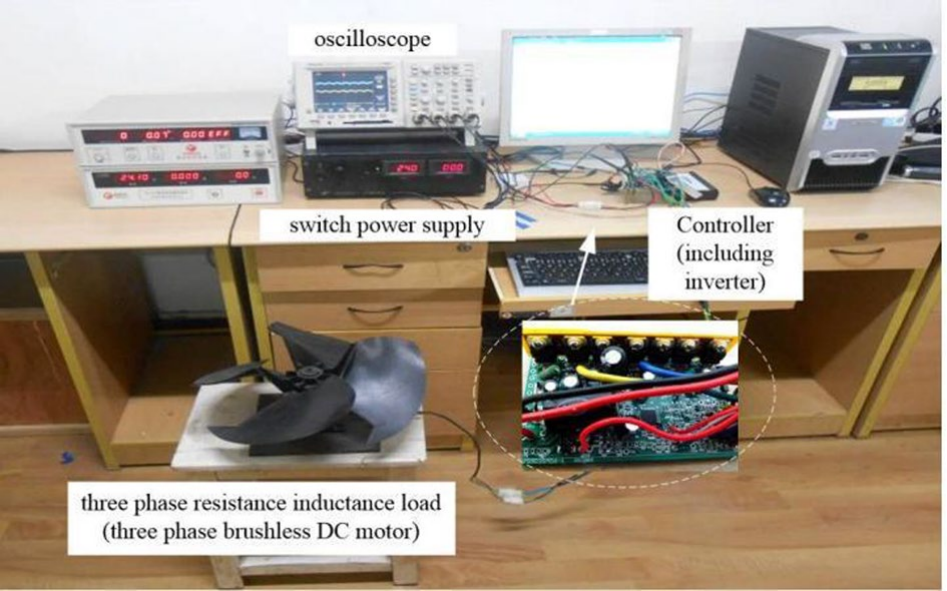
The hardware experiment platform.

**Figure 12:**
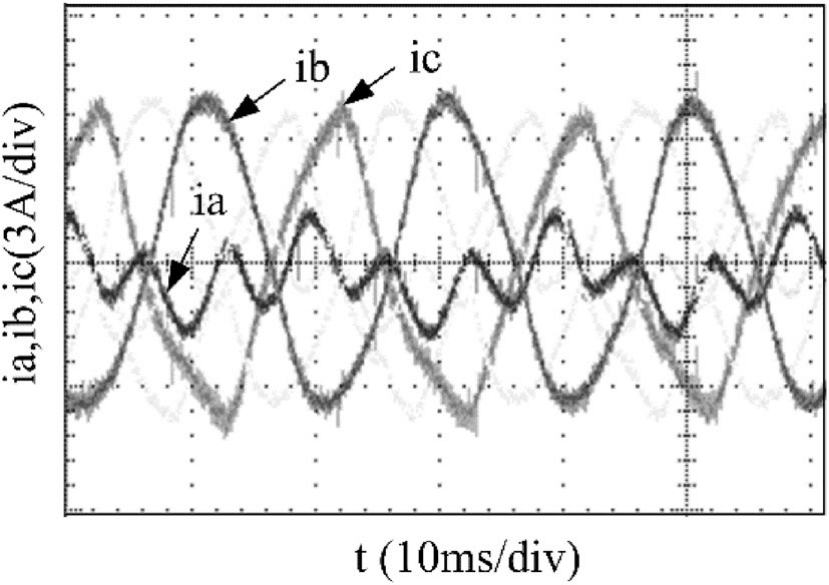
Experimental current waveforms without fault tolerant control algorithm.

**Figure 13:**
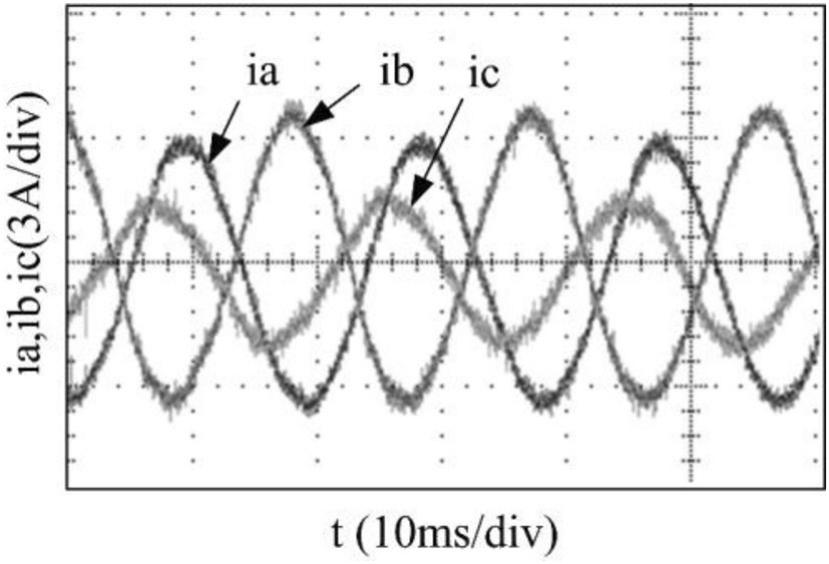
Experimental current waveforms without compensation.

**Figure 14:**
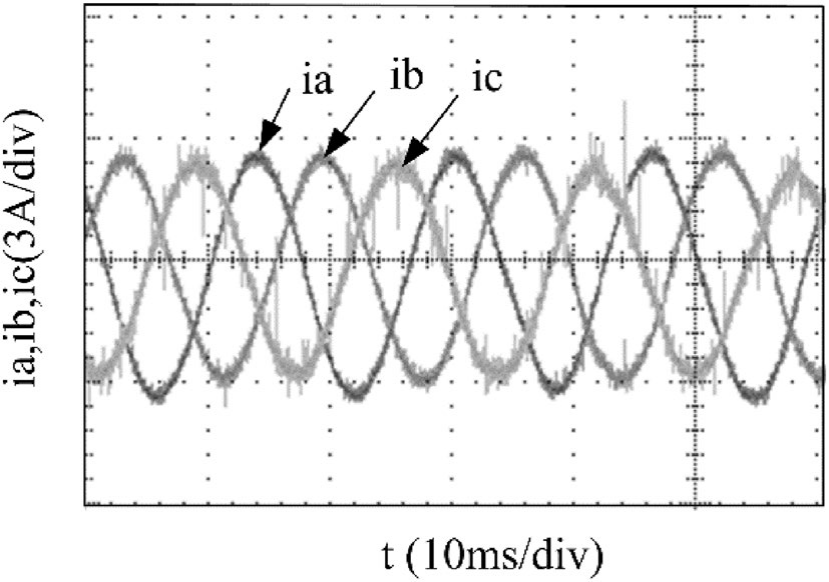
Experimental current waveforms using fault tolerant control algorithm under a phase A fault.

**Figure 15:**
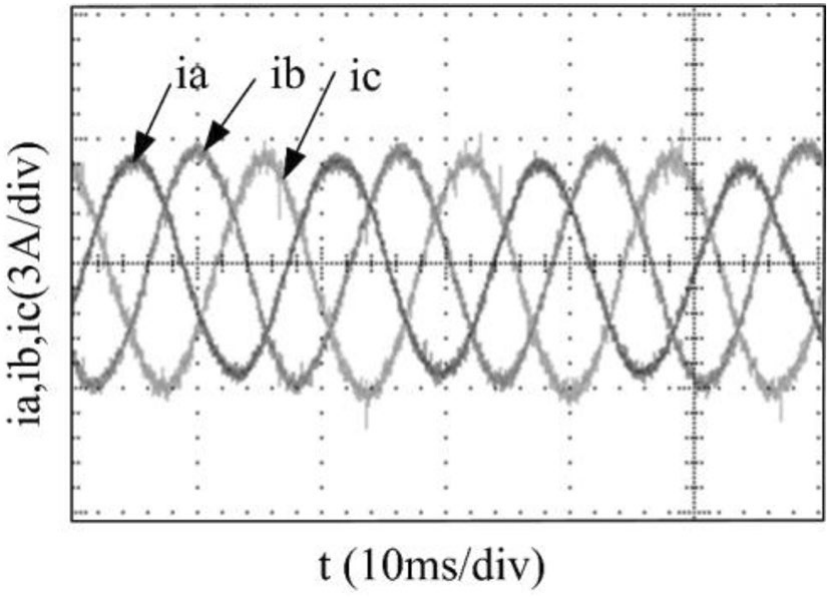
Experimental current waveforms using fault tolerant control algorithm under a phase B fault.

**Figure 16:**
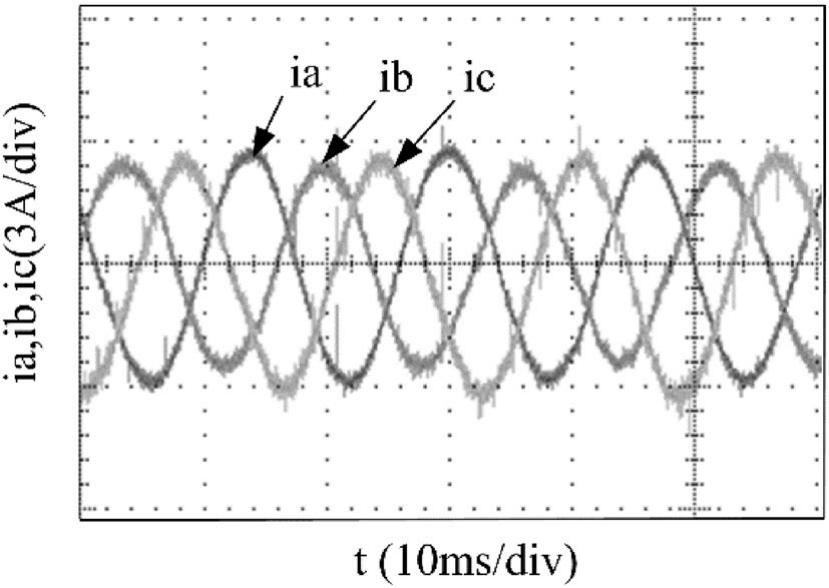
Experimental current waveforms using fault tolerant control algorithms under a phase C fault.

To further corroborate the unified SVPWM algorithm proposed in this paper, an experimental platform based on STM32F103 is built for experimental verification, as presented in Fig. 11. The experimental platform includes an inverter circuit, three phase resistance inductance load (three phase brushless DC motor), and a fan load connected to the motor shaft. The experimental parameters are the same as those in the simulation. As in Figs. 12, 13, 14, 15 and 16, the experimental results are consistent with the simulation, which also verifies the correctness and feasibility of the fault-tolerant control strategy proposed in this paper. Moreover, this approach has a certain reference value for other types of inverter fault-tolerant control.

## Conclusion

In this paper, a new fault-tolerant control strategy is proposed for the single arm fault reconstruction topology of a two-level inverter. Theoretical derivation and experimental verification are conducted, and the following conclusions are obtained.

1. Through the voltage vector coordinate transformation of the phase B and C bridge arm fault reconstruction topologies, the SVPWM algorithms for different bridge arm fault reconstruction topologies are unified. The unified SVPWM algorithm offers three advantages, which greatly reduce the complexity of fault-tolerant control. First, the sector can be judged directly according to the symbol of the $\alpha \beta$ coordinate component of the reference voltage vector, without the conventional inverse trigonometric function method. Second, the action time of the basic voltage vector can also be calculated directly according to the $\alpha \beta$ coordinate component, avoiding irrational number and trigonometric function required in conventional method of calculating the reference voltage vector modulus and phase angle. In addition, the compensation of the neutral point voltage offset in the reconstructed topology aims only at the value of $\alpha$ coordinate component, which avoids simultaneous compensation of the $\alpha \beta$ coordinate component in conventional method. The experimental results also verify the correctness and feasibility of the unified SVPWM algorithm.
2. The influence caused by the voltage offset of irrational reconstruction topology on the reference voltage vector synthesis is derived in detail, and a direct correction method based on the $\alpha$ axis component of reference voltage vector is proposed.
3. Through the algebraic operation of the transient value of three-phase current, the voltage offset is calculated and compensated for, which reduces the imbalance of the three-phase output current caused by the voltage oscillation. Compared with the traditional integral voltage compensation method, the instantaneous voltage compensation method proposed in this paper is easier to implement.

## Data Availability

The datasets generated during and/or analyzed during the current study are available from the corresponding author on reasonable request.
